# From Entry to Early Dissemination—*Toxoplasma gondii's* Initial Encounter With Its Host

**DOI:** 10.3389/fcimb.2019.00046

**Published:** 2019-03-05

**Authors:** Estefania Delgado Betancourt, Benjamin Hamid, Benedikt T. Fabian, Christian Klotz, Susanne Hartmann, Frank Seeber

**Affiliations:** ^1^FG 16: Mycotic and Parasitic Agents and Mycobacteria, Robert Koch-Institute, Berlin, Germany; ^2^Department of Veterinary Medicine, Institute of Immunology, Freie Universität Berlin, Berlin, Germany

**Keywords:** intestinal organoids, Apicomplexa, intestinal epithelial barrier, innate response, *Toxoplasma gondii*, Paneth cells

## Abstract

*Toxoplasma gondii* is a zoonotic intracellular parasite, able to infect any warm-blooded animal via ingestion of infective stages, either contained in tissue cysts or oocysts released into the environment. While immune responses during infection are well-studied, there is still limited knowledge about the very early infection events in the gut tissue after infection via the oral route. Here we briefly discuss differences in host-specific responses following infection with oocyst-derived sporozoites vs. tissue cyst-derived bradyzoites. A focus is given to innate intestinal defense mechanisms and early immune cell events that precede *T. gondii'*s dissemination in the host. We propose stem cell-derived intestinal organoids as a model to study early events of natural host-pathogen interaction. These offer several advantages such as live cell imaging and transcriptomic profiling of the earliest invasion processes. We additionally highlight the necessity of an appropriate large animal model reflecting human infection more closely than conventional infection models, to study the roles of dendritic cells and macrophages during early infection.

## Introduction

Infection by the intracellular apicomplexan parasite *Toxoplasma gondii* affects an estimated 25–30% of humans worldwide (Montoya and Liesenfeld, 2004), making this zoonotic parasite one of the most widespread human pathogens in the world. Infected felids excrete up to several hundred million environmentally resistant oocysts with their feces, which can infect any warm-blooded animal upon ingestion. There, *T. gondii* reproduces asexually via two distinct life cycle stages, the fast growing tachyzoite and the slower reproducing bradyzoite stage. The latter forms cysts in various host tissues, which may be consumed by carnivores or omnivores. Following ingestion, bradyzoites are released from cysts, reverting to the tachyzoite stage, replicating, and invading surrounding tissues before eventually disseminating throughout the body to other organs (Blader et al., 2015).

### Different Outcomes Are Observed Following Experimental Infection With Different Parasite Stages and in Different Host Species

While *T. gondii* can be transmitted via any of the above-mentioned paths, it is known that infections with different forms of the parasite have different effects in different hosts. Sporozoites differ biochemically and cell biologically from tachyzoites and bradyzoites (Speer et al., 1995; Dubey et al., 1998; Jerome et al., 1998; Fritz et al., 2012). Understanding innate immune mechanisms will therefore require comparisons of infections with oocyst-derived sporozoites and bradyzoites, as well as consideration of naturally occurring species-specific transmission pathways.

The natural predator-prey interaction of cats and rodents serves as a convincing argument for studying rats and mice as natural hosts for *T. gondii*. Experimental data (Dubey, 2001, 2006) support the hypothesis that *T. gondii* has primarily evolved for transmission by carnivory in cats and via the fecal-oral route in herbivores (Dubey, 2006). *Mus musculus* is almost entirely an herbivorous organism, with occasional insectivorism (Butet and Delettre, 2011). Maternal cannibalism, as seen under lab conditions, is rather a stress-related behavior (Weber and Olsson, 2008; Weber et al., 2013) and is presumably much less often observed in nature. Consequently, the common use of oral *T. gondii* infection with bradyzoites in mice as a research model is problematic, particularly if we wish to gain insights relevant to human infection (Ehret et al., 2017; Sher et al., 2017). Notably, islands free of felids exhibited a low seroprevalence of *T. gondii* in wild pigs and humans, likely resulting from a lack of oocysts in the environment (Dubey et al., 1997a; de Wit et al., 2019). This underlines the important role that oocysts play in parasite dissemination, even for omnivorous species such as pigs or humans.

### The Early Events of Intestinal Entry of *T. gondii*

Very little is known in any organism about the very early phase of infection with either bradyzoites or sporozoites regarding mechanisms employed by the parasite to pass through the intestinal epithelial barrier (IEB). Early *in vivo* studies reported that excysted sporozoites were observed in enterocytes 30 min post-infection, with few cytopathological lesions such as villi enlargement detected at the ultrastructural level (Dubey et al., 1997b; Speer and Dubey, 1998). Sporozoites could pass through enterocytes and goblet cells of the ileal epithelium 2 h post-infection and enter the lamina propria where parasites differentiated. However, recent reports have concluded that parasites are only reliably detectable by *in vivo* imaging 3–5 days post-infection (Coombes et al., 2013; Gregg et al., 2013).

There is therefore a clear need for cellular systems which mimic the *in vivo* situation and allow live cell imaging and transcriptomic profiling of the earliest invasion processes. Great advances in generation, cultivation and cell-type characterization of intestinal organoids (IOs) offer unique opportunities to observe these early events in different hosts with different *T. gondii* stages (Klotz et al., 2012; Derricott et al., 2019).

### Intestinal Organoid Models to Study *T. gondii* Infections

**Figure 1:**
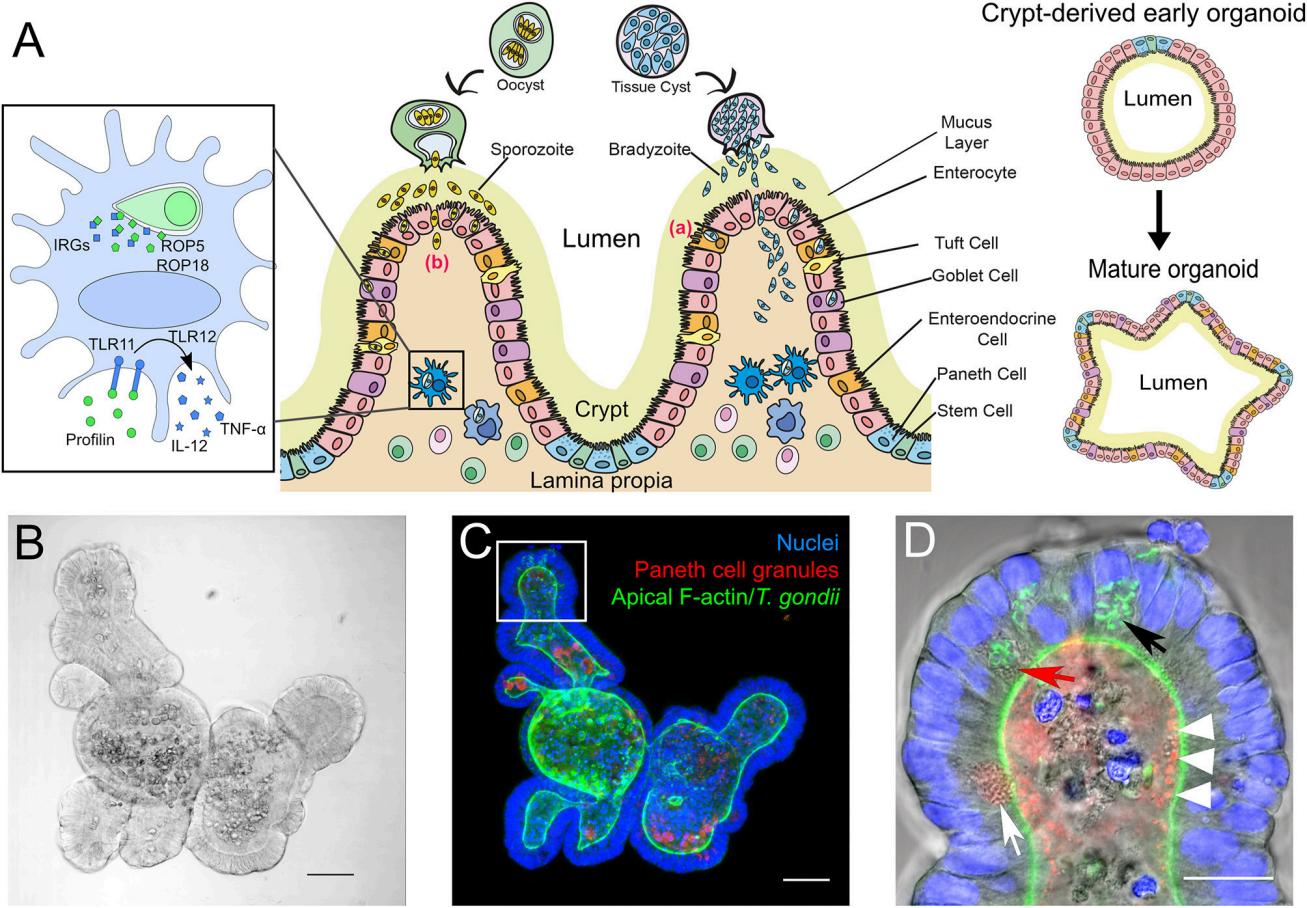
**(A)** Simplified scheme of the intestinal mucosa with its different cell populations and its derived organoids, and its infection with *T. gondii* bradyzoites or sporozoites. Center: After oral uptake of oocysts or tissue cysts into the small intestine sporozoites or bradyzoites leave the cyst and enter the intestinal epithelium to reach the lamina propria. The principle strategies used by the parasite to cross this barrier are either via invasion of the intestinal epithelial cells (a) or by transepithelial migration (b). Left: Infected murine DC. *T. gondii* ROP5 and ROP18 phosphorylate host IRGs, thus protecting the PV from degradation. Extracellular profilin is recognized by the cell via TLRs 11 and 12, inducing the release of IL-12 and TNF-α. Right: Extracellular matrix-embedded crypt (generated via physical disruption of the intestine) leads under appropriate culture conditions to the initial formation of a crypt-derived organoid which then can differentiate into a more complex “mature” intestinal organoid. **(B–D)** Microscopic images of a mouse small intestine-derived organoid infected with *T. gondii* RH strain tachyzoites. **(B)** Representative bright-field confocal image of a mouse IO after 7 days in culture. Scale bar 50 μm. **(C)** Projection of a confocal z-stack of the IO shown in **C**, stained with FITC-phalloidin for apical F-actin (green), TRITC-labeled UEA-1 lectin for Paneth cell granules (red) and DAPI for nuclei (blue). Note that due to the projection of the stack fluorescent signals might appear mis-localized within the organoid compared to the single plane shown in **(D)**. Scale bar 50 μm. **(D)** Enlarged view of an organoid villus-like structure (white square in **C**) of a single plane. Parasites (identifiable by their GFP-tagged green tubular mitochondrion (Thomsen-Zieger et al., 2003; black arrow) had replicated in IECs for 48 h. Paneth cells, identifiable by their multiple granules (white arrow) can be detected in the villus-like structure. Due to its granular appearance the red arrow indicates a possible Paneth cell containing replicating parasites. The IO's lumen is filled with cell debris from apoptotic cells, constantly shed as part of the high turnover rate of IECs. The red structures in the lumen marked with white arrow heads might indicate Paneth cell degranulation, as previously described by Farin et al. (2014). Scale bar 20 μm.

IOs can serve as an unlimited source for primary intestinal epithelial tissues. They reflect the cellular content and functionality of the *in vivo* organ (Figure 1A) including the unique properties of the IEB, like composition of tight junctions (Chiba et al., 2008; Kozuka et al., 2017). By manipulating culture conditions IOs can display the different cell populations that vary throughout both small and large intestine. A major advantage of IOs is their long-term survival *in vitro* (in contrast to *ex vivo* organ cultures) and the savings on animal experiments once IOs have been established. They are also genetically tractable (Schwank et al., 2013). However, IOs also have drawbacks, like issues with reproducibility and comparability of results between labs due to non-standardized culture conditions and/or different donors for organoid preparations (Bartfeld, 2016; Yu et al., 2017). Moreover, although IOs provide a good representation of the complexity of the intestinal tissue, terminally differentiated IOs with all cell populations, in particular the less frequent Tuft- and M-cells, are difficult to obtain. Other aspects that are missing in IOs compared to whole animals or *ex vivo* short-term organ cultures are immune cells and the microbiota. However, recent technological advances allow these “missing” components to be gradually incorporated into the system to consecutively reproduce the *in vivo* situation (Bartfeld, 2016; Hill et al., 2017; Noel et al., 2017; Williamson et al., 2018). Another hurdle, in particular for infection studies, is the inverted topology of the IOs, i.e., the apical side of the epithelial cells faces the lumen of the organoid (Figures 1C,D). This requires the pathogens to be introduced via microinjection (Bartfeld and Clevers, 2015; Hill et al., 2017; Heo et al., 2018; Williamson et al., 2018). However, in the case of *T. gondii* infection of IOs is efficiently occurring (Figures 1B–D) when its lumen becomes accessible by physically breaking it open via simple pipetting. From this short discussion it is evident that for studying *T. gondii* biology, depending on the research question, three-dimensional IOs are superior to 2D- or even 3D-cultures of cell lines (Barrila et al., 2018; Danielson et al., 2018) but that they cannot yet fully replace mouse experiments.

Intestinal epithelial cells (IECs) are constantly renewed every 3–4 days (Clevers and Bevins, 2013). The main absorptive cell type, enterocytes, are characterized by a columnar architecture and microvilli at the apical surface. Goblet cells secrete mucins, which form a thick mucus layer along the epithelial surface, limiting access and promoting removal of potential invading pathogens (Johansson and Hansson, 2016). Enteroendocrine cells release hormones at the basolateral site, mediating paracrine effects to neighboring cells (Allaire et al., 2018). Tuft cells are thought to serve as luminal chemosensors, but their main function is largely unknown (Gerbe and Jay, 2016). Paneth cells are located in the crypt base and play a pivotal role in innate immune defense in the intestine by secreting antimicrobial molecules, such as defensins/cryptidins, lysozyme and phospholipases (Cheng and Leblond, 1974; Clevers and Bevins, 2013). The release of these granules is highly dependent on several stimuli such as cholinergic agonists (Satoh et al., 1995; Clevers and Bevins, 2013). Paneth cell-specific autophagy has been shown recently as essential for protection against interferon-γ (IFN-γ) dependent intestinal inflammation and crypt integrity, also in the context of a *T. gondii* infection (Raetz et al., 2013; Burger et al., 2018). Another important role of Paneth cells is the maintenance of the stem cell niche in the small intestine (Sato et al., 2011; Clevers and Bevins, 2013).

IOs allow the real-time study of early infection event dynamics in specific gut epithelial cell types upon *T. gondii* infection. This includes the ability to study IOs derived from a range of host species, including rodents, pigs and humans (Klotz et al., 2012; Derricott et al., 2019). They can therefore serve to highlight differences in these processes in different species under comparable experimental conditions, due to the absence of the immune system and microbiota. In the past, infection studies were performed using either the murine *in vivo* model, or small intestinal cell lines derived from immortalized or cancer cells. It will therefore be crucial to compare results to *T. gondii*-infected IOs.

### Innate Defense Mechanisms of Intestinal Epithelial Cells to *T. gondii* Infection

Studies in mice have provided a comprehensive picture of innate immune responses in the lamina propria and beyond (Yarovinsky, 2014; Cohen and Denkers, 2015; Dunay and Diefenbach, 2018) upon *T. gondii* infection, as well as the role of the microbiota (Cohen and Denkers, 2014; Leung et al., 2018). However, much less is known about how parasites are able to overcome the IEB or their fate in the individual cells of it (Jones et al., 2017).

The intestinal mucosa is protected by physical barriers that include the mucus layer, the glycocalyx of enterocytes and the tight junctions between intestinal epithelial cells (Pelaseyed et al., 2014; Okumura and Takeda, 2017). It shields the mucosa from invasion by the microbiota. Although *T. gondii* is apparently able to penetrate this barrier the efficiency of this process and how the hurdles are overcome is unknown.

Few studies have addressed the role of glycocalyx and mucus during *T. gondii* infection. However, one described an increase in mucus-producing goblet cells in rats upon infection with oocysts (Trevizan et al., 2016). Conflicting results were reported for the role that trefoil factor family (TFF) peptides, major constituents of intestinal mucus, play in a *T. gondii* infection. While one study in mice showed a protective effect of TFF2 against immunopathology (McBerry et al., 2012), another reported the opposite effect for TFF3 (Fu et al., 2015).

Different pathways have been proposed to be used by *T. gondii* for transmigration into the intestinal epithelial tissue (Jones et al., 2017) (Figure 1A). The first one is paracellular transmigration, in which the parasites, aided by their gliding motility, move through the intercellular junctions without altering the barrier integrity. *In vitro* studies using intestinal polarized monolayers showed that tachyzoites of type I strains exhibited a higher migratory capacity compared to type II and type III strains. This might contribute to the higher virulence of type I strains seen in lab mice (Barragan and Sibley, 2002). It was later shown that parasites rapidly cluster between the cellular junctions upon entry (Weight and Carding, 2012; Briceño et al., 2016; Jones et al., 2017), and the tight junction protein occludin was identified as a specific target of tachyzoites during passage through the paracellular pathway (Weight et al., 2015). *T. gondii* might use it to efficiently cross the monolayers by the interaction of intracellular adhesion molecule-1 with the parasite adhesion molecule MIC2, without affecting barrier permeability (Barragan et al., 2005). However, contradictory results were reported in an experimental set-up with Caco-2 cells (Briceño et al., 2016) where it was shown that intestinal barrier function was disturbed. These examples highlight the need to evaluate these crucial events in a cellular system like IOs that closely resemble the *in vivo* IEB.

The second reported entry pathway is by penetration of the apical cell membrane and passing through the basolateral side in order to reach the underlying lamina propria where leukocytes reside (Barragan et al., 2005; Lambert and Barragan, 2010). Several authors have proposed a third pathway, which involves a Trojan-horse-like model (Gregg et al., 2013; Jones et al., 2017). Upon infection of IECs neutrophils are rapidly recruited to the site of infection and subsequently infected by the parasite. These are then capable of migrating through the epithelial cell layer and crossing the lumen, thereby facilitating parasite spread not only in the intestine but also to other tissues (Coombes et al., 2013). However, most of these data were generated in the above-mentioned traditional model systems. Therefore, it will be interesting to see how IOs compare to these models and which reflect best the *in vivo* situation.

Besides the physical barrier discussed above an independent biochemical barrier exists, composed mainly of antimicrobial peptides and proteins (e.g., cryptidins, defensins, lysozyme). The vulnerability of *T. gondii* bradyzoites or sporozoites toward these molecules is largely unknown. A differential effect of oocysts of different genetic backgrounds on Paneth cell-derived lysozyme expression following infection of BALB/c mice was reported (Lu et al., 2018). Likewise, only a single study provided some indirect evidence for an effect of the antimicrobial activity of cryptidins on bradyzoites in the lumen of mice prior to invasion of the epithelial layer (Foureau et al., 2010). Evidently, there is still more to learn in this area.

Upon parasite exposure IECs (and immune cells, see below) recognize the parasite through pattern recognition receptors on the cell surface, such as Toll-like receptors (TLRs), which activate secretion of pro-inflammatory cytokines that induce a subsequent Th1 response (Gopal et al., 2008). Among these, Paneth cell-resident TLR9 has been shown to modulate recognition of external pathogens and to induce the immune response through mechanisms such as defensin release (Rumio et al., 2004; Buzoni-Gatel et al., 2006; Foureau et al., 2010). Surprisingly, a recent transcriptomic study with rat intestinal IEC-18 cells did not find evidence of pathogen-associated molecular patterns being induced upon infection with oocyst-derived *T. gondii* sporozoites (Guiton et al., 2017).

### Early Interaction of *T. gondii* With Host Immune Cells

The lamina propria and Peyer's patches are rich in dendritic cells (DCs) and macrophages (MΦs). Once the intestinal barrier is overcome by *T. gondii*, these are the first immune cells to recognize parasite infection and to initiate the mounting of the host immune response. This recognition can occur via at least three distinct routes. (1) DCs and MΦs directly phagocytose free, opsonized parasites upon crossing the epithelial barrier. (2) Both cell types also phagocytose infected apoptotic IECs (Buzoni-Gatel and Werts, 2006). (3) DCs are able to elongate through the tight junctions of the epithelium, and in mice recognize the soluble *T. gondii* antigen profilin in the lumen via TLR 11 and 12 (Yarovinsky et al., 2005; Koblansky et al., 2013). DCs and MΦs exhibit directly toxoplasmacidal effects to phagocytosed parasites. However, once stimulated they also begin to secrete interleukin (IL) 12 and tumor necrosis factor α (TNF-α) (Buzoni-Gatel and Werts, 2006). IL-12 and TNF-α induce the differentiation of CD4^+^ T cells into Th1 cells, which secrete IFN-γ. In parallel, IL-12, along with IL-15 secreted by infected IECs, stimulate natural killer (NK) cells and CD8^+^ T cells to begin secreting IFN-γ, the primary mediator of resistance to *T. gondii* (Suzuki et al., 1988). This leads to containment of the parasite and its conversion into the bradyzoite form, thereby hiding from the immune system (Hunter and Sibley, 2012; Ahmed et al., 2017).

### Role of *T. gondii* Rhoptry Proteins in Early Immune Cell Modification

One mechanism by which IFN-γ mediates parasite destruction in mice is through upregulation of immunity-related GTPases (IRGs) (Gazzinelli et al., 2014; Müller and Howard, 2016). IRGs are intracellular host proteins, some of which localize to the PV membrane in an infected cell causing membrane rupture, parasite release into the host cell cytosol and its subsequent degradation. In order to avoid destruction, *T. gondii* has evolved a means to subvert this host defense mechanism (Buzoni-Gatel and Werts, 2006; Gazzinelli et al., 2014). It is dependent on several parasite proteins which are derived from unique secretory organelles (rhoptries and dense granules) and transported into the infected cell. ROP18 is able to phosphorylate host IRGs such as Irga6 while ROP5 modulates this activity, all eventually resulting in PV membrane destruction (Reese et al., 2011; Behnke et al., 2012; Fleckenstein et al., 2012; Niedelman et al., 2012; Etheridge et al., 2014). However, depending on the genetic background of the mouse, this virulence mechanism of the parasite can be overcome by a highly polymorphic IRG protein (Irgb2-b1). Some of its variants can act as decoys for ROP5/18 binding, enabling other IRGs to degrade the PV (Lilue et al., 2013). Surprisingly, it is unknown if or to what extent this IRG response plays a role in the intestine. Mouse IOs could be very useful to shed light on this immediate obstacle the parasite has to overcome in order to proliferate and disseminate.

### Phenotypic Changes of DCs Utilized as “Trojan Horse” Vehicles for Dissemination of *T. gondii*

After infection *T. gondii* is able to rapidly disseminate throughout the body. Within hours it is found in the spleen and it is also able to cross the blood-brain barrier, the placental barrier in pregnant hosts, and enter immune privileged sites such as the eyes (Lambert and Barragan, 2010; Harker et al., 2015). This is achieved through invasion and utilization of migratory leukocytes as “Trojan horses.” There is evidence suggesting that several cell types may be used for this purpose, including DCs, MΦs, neutrophils, NK cells, and T cells (Courret et al., 2006). However, extracellular tachyzoites released from infected endothelial cells in the brain vasculature have also recently been implicated in overcoming the blood-brain barrier (Konradt et al., 2016).

Mostly DCs, inherently able to become migratory, have been implicated in early dissemination (Weidner and Barragan, 2014; Kanatani et al., 2015; Brasil et al., 2017; Ólafsson et al., 2018). Following activation by an antigen they undergo a series of phenotypic changes required for its efficient presentation. This includes comprehensive remodeling of the actin cytoskeleton and the loss of actin-rich structures called podosomes. These changes are essential for switching from a strongly adhesive to a migratory phenotype, allowing cells to reach the lymph nodes. Using lipopolysaccharide to activate DCs indicated that remodeling was dependent on TLR4 signaling and prostaglandin E2 (PGE2) secretion (van Helden et al., 2010; Weidner et al., 2013). In contrast, following infection with *T. gondii* tachyzoites, phenotypic changes occurred < 10 min post-invasion and were not reliant on TLR4 or PGE2. This was shown experimentally to require active manipulation by live *T. gondii* (Weidner et al., 2013). Recent studies indicated that *T. gondii* infection results in a marked reduction in pericellular proteolytic activity by DCs, mediated via the release of tissue inhibitor of metalloproteinase 1. This suggests a compensatory mechanism for an upregulation of matrix metalloproteinases, which have been demonstrated to perform diverse catalytic and non-catalytic functions in amoeboid migration (Orgaz et al., 2014; Ólafsson et al., 2018).

### Differences in the Macrophage/DC Responses of Mice and Humans to *T. gondii*: the Pig as Human-Relevant Model

As discussed, murine DCs are able to undergo maturation in response to the detection of the soluble *T. gondii* antigen profilin via TLR11 and TLR12. However, in humans TLR12 is entirely absent, and TLR11 is apparently a non-functional pseudogene (Zhang et al., 2004; Roach et al., 2005; Ishii et al., 2008). Consequently, profilin does not elicit an immune response in humans; instead it relies on phagocytosis of tachyzoites (Tosh et al., 2016; Sher et al., 2017). Although the pattern recognition receptors responsible for the recognition of *T. gondii* in humans have not been definitively identified, human PBMCs produce pro-inflammatory cytokines following stimulation with *T. gondii* RNA or DNA. This implicates the involvement of TLRs 7, 8, and 9 which are responsible for the recognition of nucleic acids from pathogens (Forsbach et al., 2008; Andrade et al., 2013; Jennes et al., 2017). The specific subsets of monocytes and DCs secreting IL-12 in response to *T. gondii* also differ between mice and humans. In mice, inflammatory monocytes and CD8α^+^ DCs respond, whereas the human analogs—classical monocytes and the cDC2 subset—do not. In contrast, human non-classical and intermediate monocytes and the cDC1 subset produce IL-12, which are analogous to murine patrolling monocytes and CD8α^−^ DCs (Tosh et al., 2016; Sher et al., 2017).

There is a clear need for an immunologically more human-like large animal model to understand the mechanisms underlying *T. gondii* infection and immunity in humans (Ahmed et al., 2017; Sher et al., 2017). The pig is one such candidate that could be utilized for this purpose. Genomic studies have indicated that 80% of porcine immune response genes resemble human equivalents, whereas for mice <10% are similar (Meurens et al., 2012; Mair et al., 2014) (Table 1). Of particular note is that like humans, pigs lack TLRs 11 and 12 (Uenishi and Shinkai, 2009; Mair et al., 2014) and so are presumably also unable to respond to profilin. They do however exhibit TLRs 7, 8, and 9, and so are likely able to recognize *T. gondii* via the same mechanism as humans (Uenishi et al., 2012; Jennes et al., 2017). Thus, the initial porcine DC and MΦ responses to *T. gondii* deserve further examination regarding their similarity to the human response.

**Table 1:** Markers of monocyte and DC subsets in mice, humans, and pigs (Fairbairn et al., 2013; Summerfield et al., 2015; Sher et al., 2017).

**Monocytes**				**Dendritic cells**			
	**Mouse**	**Human**	**Pig**		**Mouse**	**Human**	**Pig**
Classical/Inflammatory	CD11b^+^ CD115^+^ Ly6C high	HLA-DR^+^ CD14^+^ CD16^−^	CD172a^+^ CD14 high CD16 low CD163 low	cDC1	CD11c^+^ MHC II^+^ CD8a^−^ CD11b^+^	CD11c^+^ HLA-DR^+^ CD1c^+^ CD141^−^	CD135^+^ wCD11R1^+^ CD1a^+^ CD172a^+^
Intermediate		HLA-DR^+^ CD14^+^ CD16^+^	CD172a^+^ CD14 low CD16 high CD163 high	cDC2	CD11c^+^ MHC II^+^ CD8a^+^ CD11b^−^	CD11c^+^ HLA-DR^+^ CD1c^−^ CD141^+^	CD135^+^ wCD11R1^+^ CD1a^−^ CD172a^−^/low
Non-Classical/Patrolling	CD11b^+^ CD115^+^ Ly6C low	HLA-DR^+^ CD14 low CD16^+^					

*Murine and human subsets highlighted in yellow produce IL-12 in response to stimulation with T. gondii tachyzoites in vitro (Sher et al., 2017). The porcine subsets which respond to T. gondii exposure are currently unknown*.

There are also clinical similarities between the human and porcine responses to *T. gondii*, which further suggest the pig may be an appropriate model for human infection. For example, postnatal infection with *T. gondii* is usually asymptomatic or mild in humans and pigs, whereas infections with some parasite strains can be fatal in mice (Dubey, 1986; Nau et al., 2017). During pregnancy in humans and pigs parasites can often cross the placental barrier and result in abortion or congenital toxoplasmosis (Jungersen et al., 2001), whereas fetal infections are rare in immunocompetent mice (Shiono et al., 2007; Nau et al., 2017). Furthermore, as omnivorous mammals pigs, like humans, are naturally at risk of exposure to both *T. gondii* tissue cysts and oocysts in their diet (Meurens et al., 2012). This makes them a more natural host for research into the early stages of infection with both bradyzoites and sporozoites.

Although fewer immunological reagents are currently available for swine in comparison to mice and humans, this is an area undergoing rapid progress, not the least because of the increased interest in pig organs for xenotransplantation (Meier et al., 2018). After mice and primates, the porcine immune system is perhaps the next most thoroughly characterized, with pigs being firmly established as a model organism for infection research. This includes their use as a model for infection with other human-relevant, orally-acquired pathogens such as *Helicobacter pylori* and human rotavirus, as well as the protozoan parasite *Cryptosporidium parvum* (Meurens et al., 2012). In recent years the body of literature on the porcine cellular immune response specifically to *T. gondii* also increased (e.g., Miranda et al., 2015; Jennes et al., 2017; Nau et al., 2017). Notably, porcine IOs have also been described recently (Derricott et al., 2019).

## Concluding Remarks

IOs closely resemble the *in vivo* intestinal barrier and represent a source of species-specific IECs. To mechanistically study interactions of pathogens with such a complex organ it is advantageous to examine the contribution of individual epithelial cells in the absence of immune cells and microbiota. However, several reports have illustrated that IOs can be co-cultured with DCs, MΦs, IELS (Nozaki et al., 2016; Noel et al., 2017; Ihara et al., 2018; Nakamura, 2018) and also with bacteria (Hill et al., 2017; Williamson et al., 2018), thereby complementing this system as required.

The pig allows for tissue-specific translational research since the immune parameters depicted so far closely resemble humans. Future studies will show whether porcine intestinal innate and adaptive parameters better reflect human early infection events in comparison to mice.

## Ethics Statement

The use of animal material was approved by the responsible local authorities of the German Federal State Berlin (permit T0173/14).

## Author Contributions

ED provided parts of Figure 1A and Figures 1B–D. BH provided parts of Figure 1A. All authors contributed to the text and approved its final version.

### Conflict of Interest Statement

The authors declare that the research was conducted in the absence of any commercial or financial relationships that could be construed as a potential conflict of interest.
